# Diagnostic performance of Glial Fibrillary Acidic Protein and Prehospital Stroke Scale for identification of stroke and stroke subtypes in an unselected patient cohort with symptom onset < 4.5 h

**DOI:** 10.1186/s13049-022-01065-7

**Published:** 2023-01-05

**Authors:** Henriette S. Jæger, Ditte Tranberg, Karianne Larsen, Jan B. Valentin, Rolf A. Blauenfeldt, Sebastian Luger, Kristi G. Bache, Martin F. Gude

**Affiliations:** 1 The Norwegian Air Ambulance Foundation, Research and Development, Oslo, Norway; 2 Institute of Basic Medical Sciences, University of Oslo, Oslo, Norway; 3 Department of Research and Development, Prehospital Emergency Medical Services, Central Denmark Region and Aarhus University, Olof Palmes Allé 34, 2., 8200 Aarhus N, Denmark; 4 Danish Center for Clinical Health Services Research (DACS), Department of Clinical Medicine, Aalborg University and Aalborg University Hospital, Aalborg, Denmark; 5 Department of Neurology and Danish Stroke Center, Aarhus University Hospital, 8200 Aarhus, Denmark; 6 Department of Neurology, University Hospital Frankfurt/Goethe University Frankfurt, Frankfurt am Main, Germany; 7 Research and Dissemination, Østfold University College, Halden, Norway

**Keywords:** Stroke, Diagnosis, GFAP, PreSS, Prehospital

## Abstract

**Introduction:**

Rapid identification and treatment of stroke is crucial for the outcome of the patient. We aimed to determine the performance of glial fibrillary acidic protein (GFAP) independently and in combination with the Prehospital Stroke Score (PreSS) for identification and differentiation of acute stroke within 4.5 h after symptom onset.

**Patients and methods:**

Clinical data and serum samples were collected from the Treat-Norwegian Acute Stroke Prehospital Project (Treat-NASPP). Patients with suspected stroke and symptoms lasting ≤ 4.5 h had blood samples collected and were evaluated with the National Institutes of Health Stroke Scale prospectively. In this sub study, NIHSS was retrospectively translated into PreSS and GFAP was measured using the sensitive single molecule array (SIMOA).

**Results:**

A total of 299 patients with suspected stroke were recruited from Treat-NASPP and included in this study (44% acute ischemic stroke (AIS), 10% intracranial hemorrhage (ICrH), 7% transient ischemic attack (TIA), and 38% stroke mimics). ICrH was identified with a cross-fold validated area under the receiver-operating characteristic curve (AUC) of 0.73 (95% CI 0.62–0.84). A decision tree with PreSS and GFAP combined, first identified patients with a low probability of stroke. Subsequently, GFAP detected patients with ICrH with a 25.0% sensitivity (95% CI 11.5–43.4) and 100.0% specificity (95% CI 98.6–100.0). Lastly, patients with large-vessel occlusion (LVO) were detected with a 55.6% sensitivity (95% CI 35.3–74.5) and 82.4% specificity (95% CI 77.3–86.7).

**Conclusion:**

In unselected patients with suspected stroke, GFAP alone identified ICrH. Combined in a decision tree, GFAP and PreSS identified subgroups with high proportions of stroke mimics, ICrH, LVO, and AIS (non-LVO strokes).

## Introduction

Stroke is a major cause of mortality and morbidity worldwide [1]. Symptoms of ischemic and hemorrhagic strokes are similar, but require different treatment strategies. Rapid initiation of correct treatment is crucial for patient outcome [2, 3].

Reperfusion therapy, by intravenous tissue plasminogen activator (tPA) and/or endovascular treatment (EVT) in patients with large vessel occlusion (LVO), has greatly improved patient outcomes after acute ischemic stroke (AIS) [2, 4–6]. Patients suspected of AIS are recommended to be transported to the nearest hospital for rapid tPA administration [7]. EVT is offered at comprehensive stroke centers (CSC) but the best transport strategy for patients with LVO remains unknown [7–9]. Interhospital transfers from a primary stroke center (PSC) to a CSC is associated with EVT delay and may cause poorer outcomes [10]. To comply with any transport strategy for patients with LVO, early prehospital identification is pivotal [7]. Shortened versions of the NIHSS are used for prehospital stroke screening and severity assessment to predict AIS caused by LVO [11–13]. In this study, we used the two-part stroke scale Prehospital Stroke Scale (PreSS) derived from National Institutes of Health Stroke Scales (NIHSS) for stroke screening and LVO identification [14]. All stroke scales based on NIHSS for LVO identification seem to reach a ceiling effect with areas under the receiver-operating curve (AUCs) of up to 0.70–0.85 [12, 13]. Additional tools are needed to increase prehospital stroke subtype identification.

Glial fibrillary acidic protein (GFAP)—a brain-specific protein—is released to the blood stream after brain tissue damage [15]. A significant difference in serum GFAP levels has been shown between intracerebral hemorrhage (ICH), AIS, and stroke mimics within 2–6 h after symptom onset [16–20]. GFAP levels are associated with stroke severity, infarct volumes, bleeding volumes [20–22], and stroke location [21, 23]. Serum levels of GFAP are low/undetectable in individuals without stroke [16, 18, 23].

The new SIMOA technology measures GFAP with a higher sensitivity than conventional enzyme-linked immunosorbent assay (ELISA) [24]. Values under the limit of detection using conventional ELISA can be obtained with SIMOA [25].

In a previous study, the addition of ELISA-measured prehospital biomarkers to a stroke scale was only associated with a modest increase in performance [26].

Our objective was to investigate the ability of GFAP and PreSS to identify stroke subgroups in an unselected patient cohort with suspected stroke. Using SIMOA, we investigated the ability of GFAP to identify ICrH and further, organized in a decision tree, the ability of GFAP combined with PreSS to identify groups of patients, i.e., separating stroke mimics, ICrH, LVO, and AIS (non-LVO strokes).

## Methods

### Standard protocol approvals, registrations, and patient consents

This study used NIHSS scores and blood samples collected in the Treat-Norwegian Acute Stroke Prehospital Project (Treat-NASPP) [27]. Treat-NASPP was approved by the Regional Committees for Medical and Health Research Ethics (document-ID Treat-NASPP: 2016/974). Consent was collected from all included patients, or their legal representative, in the acute phase, during their hospital stay, or after discharge.

### Study design

This study was designed as a diagnostic sub study to Treat-NASPP. Prospectively collected blood samples and clinical data from the trial were used. We included patients that were assessed with the full NIHSS and had blood samples collected in MSU/ambulance or at hospital admission. The anesthesiologists and paramedics working in the MSU/ambulance were trained in blood collection and NIHSS assessment through a 2-day theoretical and practical training course [27, 28].

### Study population

**Fig. 1:**
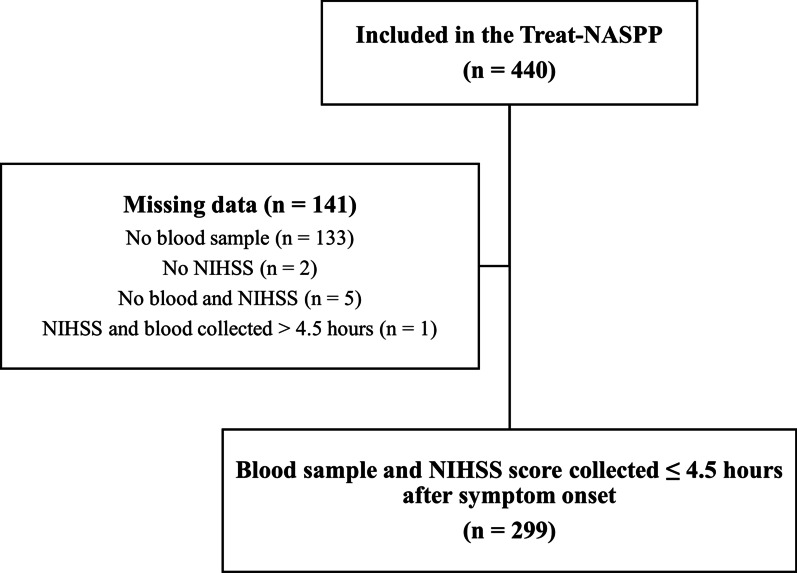
Study population. Abbreviations: *NIHSS* National Institutes of Health Stroke Scale, *Treat-NASPP* Treat Norwegian Acute Stroke Prehospital Project

The Treat-NASPP was conducted in Østfold County, Norway, from May 2017 throughout March 2020. Inclusion of patients to Treat-NASPP has been described elsewhere [27]. Patients included in this sub study had been enrolled in Treat-NASPP with ongoing stroke symptoms lasting ≤ 4.5 h, an assessment with NIHSS and blood samples had been collected. This took place in the MSU, regular ambulance by specially trained paramedics, or upon hospital arrival. Most patients included in the ordinary ambulances were assessed by paramedics not trained in NIHSS or blood sampling which was the main reason for missing blood samples and NIHSS and exclusion from this sub study (Fig. 1).

### Inclusion criteria

Nonpregnant suspected acute stroke patients ≥ 18 years of age with a full NIHSS and blood samples drawn within 4.5 h after symptom onset.

### Exclusion criteria

Patients < 18 years of age, missing NIHSS, missing blood samples or blood sampling later than 4.5 h after symptom onset.

### Final stroke diagnosis

Stroke is caused by ischemia (AIS) or hemorrhage (ICH or subarachnoid hemorrhage (SAH)) [29, 30]. AIS was defined as (1) infarction on brain imaging (CT/MRI) up to 3 days after symptom onset, (2) LVO on CT angiography in the acute phase, or (3) patients without infarction on brain images but with symptoms corresponding to stroke and who were initially treated with tPA.

LVO was defined as occluded intracranial internal carotid artery (ICA), middle cerebral artery (M1/M2), proximal anterior cerebral artery (A1/A2), intracranial vertebral artery (VA), basilar artery (BA), or proximal posterior cerebral artery (P1/P2) [31]. Hemorrhagic stroke was defined as non-traumatic bleeding in the brain parenchyma, ventricular system (ICH), or bleeding in the subarachnoid space (SAH) [29]. The term ICrH was defined as hemorrhagic strokes (ICH/SAH) and subdural hemorrhages (SDH) due to absolute contraindications against tPA treatment.

TIA was defined by remission of symptoms within 24 h without infarction on brain imaging 1–3 days after symptom onset. Stroke mimics were classified as patients with a final International Classification of Diseases, tenth version (ICD-10) [32] diagnosis other than I.60 (ICH), I.61 (SAH), I.62 (SDH), I.63 (AIS), and G.45 (TIA).

### Blood sampling and GFAP measurements

Blood samples were drawn within 4.5 h after symptom onset/last seen well. Venous blood was collected in 10 mL clot activator (BD, USA) serum tubes. For most patients the tubes were centrifuged (1500 G, 10 min, room temperature) within two hours after collection. For the patients that were transported directly to the CSC, the time from sample collection to centrifugation was within three hours. The difference in time from sample collection to centrifugation does not affect the GFAP levels in the samples as they are stored at room temperature and centrifuged within 24 h [33]. The serum samples were aliquoted and frozen at − 80 °C for long-term storage. The samples were not thawed until analysis.

In December 2020, the samples were sent on dry ice to Quanterix (Billerica, Massachusetts, USA) for GFAP measurements using SIMOA bead technology on a fully automated HD-1 analyzer. The time from the beginning of the analysis to the first result was about 30 min and thereafter a new result was generated every 45 s [34]. The analyses were performed by personnel blinded to the clinical information. The calibrator was analyzed in triplicates, and the control and serum samples were analyzed in duplicates. Samples were analyzed in a four-fold dilution. Samples with GFAP levels above the upper limit of detection at first analysis were reanalyzed in a 16-fold and 160-fold dilution. All samples had GFAP levels above the limit of detection (0.211 pg/mL). Upper limit depended on the sufficient dilution.

### Retrospective construction of PreSS from initial NIHSS

**Fig. 2:**
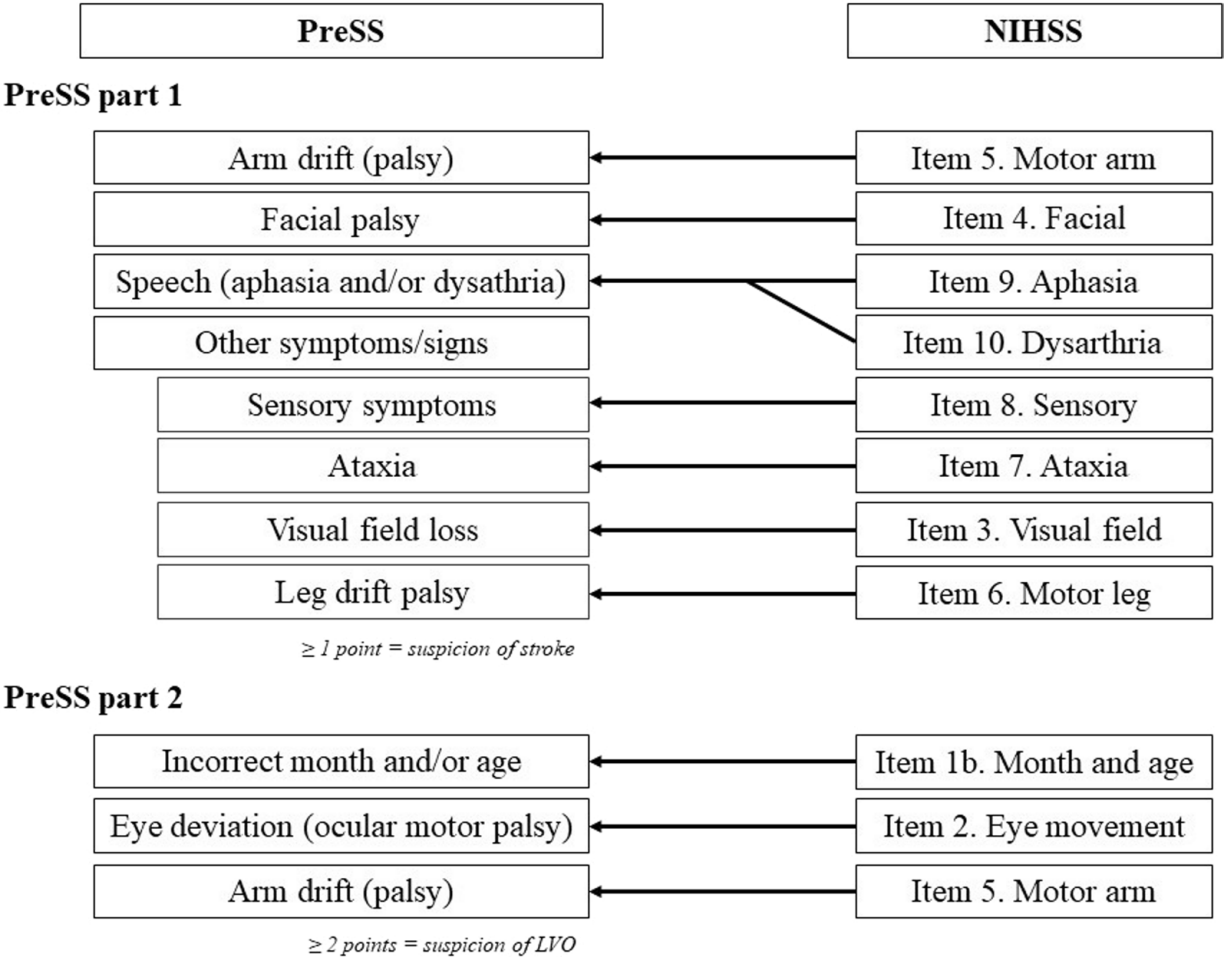
Translation of NIHSS to PreSS. Abbreviations: *LVO* large-vessel occlusion, *NIHSS* National Institutes of Health Stroke Scale, *PreSS* Prehospital Stroke Score

The NIHSS score was assessed at patient inclusion and retrospectively translated into PreSS using the principle shown in Fig. 2. Each symptom was graded 1 point independently of severity. PreSS part 1 was positive for scores of ≥ 1 point. If PreSS part 1 was positive, PreSS part 2 was performed. PreSS part 2 was positive for scores of ≥ 2 points.

### Statistical analysis

Stata version 16 (StataCorp. 2019, Stata Statistical Software: Release 16*.* College Station, TX: StataCorp LLC) was used for all statistical analyses. The performance of GFAP and PreSS was examined combined and separately to identify stroke and stroke subtypes using five-fold cross validations and logistic regression analyses. AUCs with 95% CI were determined. This was followed by calculation of sensitivity, specificity, positive predictive value (PPV), negative predictive value (NPV), positive likelihood ratio (LR+), and negative likelihood ratio (LR−). We created a decision tree for identification of stroke mimics and stroke subtypes combining PreSS and GFAP.

The GFAP cut-offs were determined as the lowest possible cut-offs yielding 100% and 95% specificity for ICrH identification.

A sensitivity analysis was performed using Cincinnati Prehospital Stroke Scale (CPSS) and NIHSS for comparison.

For baseline characteristics, variables are presented as sums, mean (standard deviation (SD)) or median (interquartile range (IQR)). Percentages were calculated based on non-missing data.

## Results

The Treat-NASPP trial included 440 patients among whom 141 were excluded due to missing blood samples (n = 133), missing NIHSS (n = 2), missing blood sample and NIHSS (n = 5), or late blood sampling > 4.5 h (n = 1) (Fig. 1). The study population consisted of 299 patients with clinical suspicion of stroke. The final diagnoses were distributed as follows: 44% AIS (n = 131), 7% ICH (n = 21), 1% SAH (n = 4), 2% SDH (n = 7), 7% TIA (n = 22), and 38% stroke mimics (n = 114). All patients had blood samples drawn in conjunction with an NIHSS assessment either in the MSU/ambulance (n = 248 (83%)) or upon arrival at hospital (n = 51 (17%)). Males were overrepresented among patients with SDH (100%), SAH (75%), and TIA (73%). The median NIHSS scores were higher for patients with ICH (12 (IQR 7;15)) and SAH (9 (IQR 4;14)) than for patients with AIS (4 (IQR 3;7)), TIA (4 (IQR 1;5)), SDH (4 (IQR 3;10)), and stroke mimics (3 (IQR 1;5)). The median time from symptom onset/last seen well to time for blood sampling was 77 (IQR 55;130) minutes in the entire cohort.

Among the 131 patients with AIS, 27 (21%) had LVO (M1 (n = 15), M2 (n = 6), ICA (n = 4), and BA (n = 2)) (Table 1).

**Table 1:** Baseline characteristics

	AIS	ICH	SAH	SDH	TIA	Stroke mimics
	N = 131	N = 21	N = 4	N = 7	N = 22	N = 114
Age at symptom onset, years, mean (SD)	73 (± 13)	70 (± 13)	67 (± 15)	80 (± 9)	74 (± 13)	65 (± 17)
Sex, female, n (%)	56 (43%)	8 (38%)	1 (25%)	0 (0%)	6 (27%)	56 (49%)
Minutes from ictus to blood collection, median (IQR)	80 (56–126)	50 (40–85)	141 (95–201)	76 (67–160)	78 (59–100)	78 (50–140)
NIHSS at blood collection, median (IQR)	4 (3–7)	12 (7–15)	9 (4–24)	4 (3–10)	4 (1–5)	3 (1–5)
GFAP (pg/mL), median (IQR)	268 (171–514)	1484 (310–5370)	718 (381–12,496)	585 (360–897)	212 (139–481)	192 (109–386)
PreSS part 1 positive, n (%)	127 (97%)	21 (100%)	4 (100%)	6 (86%)	16 (73%)	94 (82%)
PreSS part 2 positive, n (%)	32 (25%)	13 (62%)	2 (50%)	4 (67%)	2 (13%)	17 (18%)
Prehospital inclusion, n (%)	106 (81%)	15 (71%)	4 (100%)	6 (86%)	17 (77%)	100 (88%)
Treated with tPA, n (%)	110 (84%)	0 (0%)	0 (0%)	0 (0%)	0 (0%)	33 (29%)
Treated with EVT, n (%)	10 (8%)	0 (0%)	0 (0%)	0 (0%)	0 (0%)	0 (0%)
*Comorbidities*						
Atrial fibrillation, n (%)	19 (15%)	4 (20%)	0 (0%)	2 (29%)	5 (23%)	15 (13%)
Diabetes, n (%)	17 (13%)	4 (20%)	0 (0%)	1 (14%)	7 (32%)	17 (15%)
Heart disease, n (%)	48 (37%)	7 (33%)	1 (25%)	4 (57%)	14 (64%)	27 (24%)
Hypertension, n (%)	75 (57%)	16 (76%)	1 (25%)	4 (57%)	18 (82%)	55 (49%)
Hyperlipidemia, n (%)	33 (25%)	5 (25%)	1 (25%)	1 (14%)	6 (29%)	16 (15%)
Smoking^a^, n (%)	23 (23%)	1 (8%)	0 (0%)	0 (0%)	2 (11%)	17 (20%)
Obesity, n (%)	10 (18%)	3 (30%)	0 (0%)	0 (0%)	1 (13%)	6 (11%)
Previous AIS, TIA or ICH, n (%)	30 (23%)	7 (33%)	1 (25%)	2 (29%)	9 (41%)	37 (33%)
*Pathology*						
LVO, n (%)	27 (21%)					

*AIS* acute ischemic stroke, *EVT* endovascular treatment, *GFAP* glial fibrillary acidic protein, *ICH* intracerebral hemorrhage, *LVO* large-vessel occlusion, *NIHSS* National Institutes of Health Stroke Scale, *PreSS* Prehospital Stroke Score, *SAH* subarachnoid hemorrhage, *SDH* subdural hematoma, *TIA* Transient ischemic attack, *tPA* tissue plasminogen activator

^a^Smoking at inclusion

**Fig. 3:**
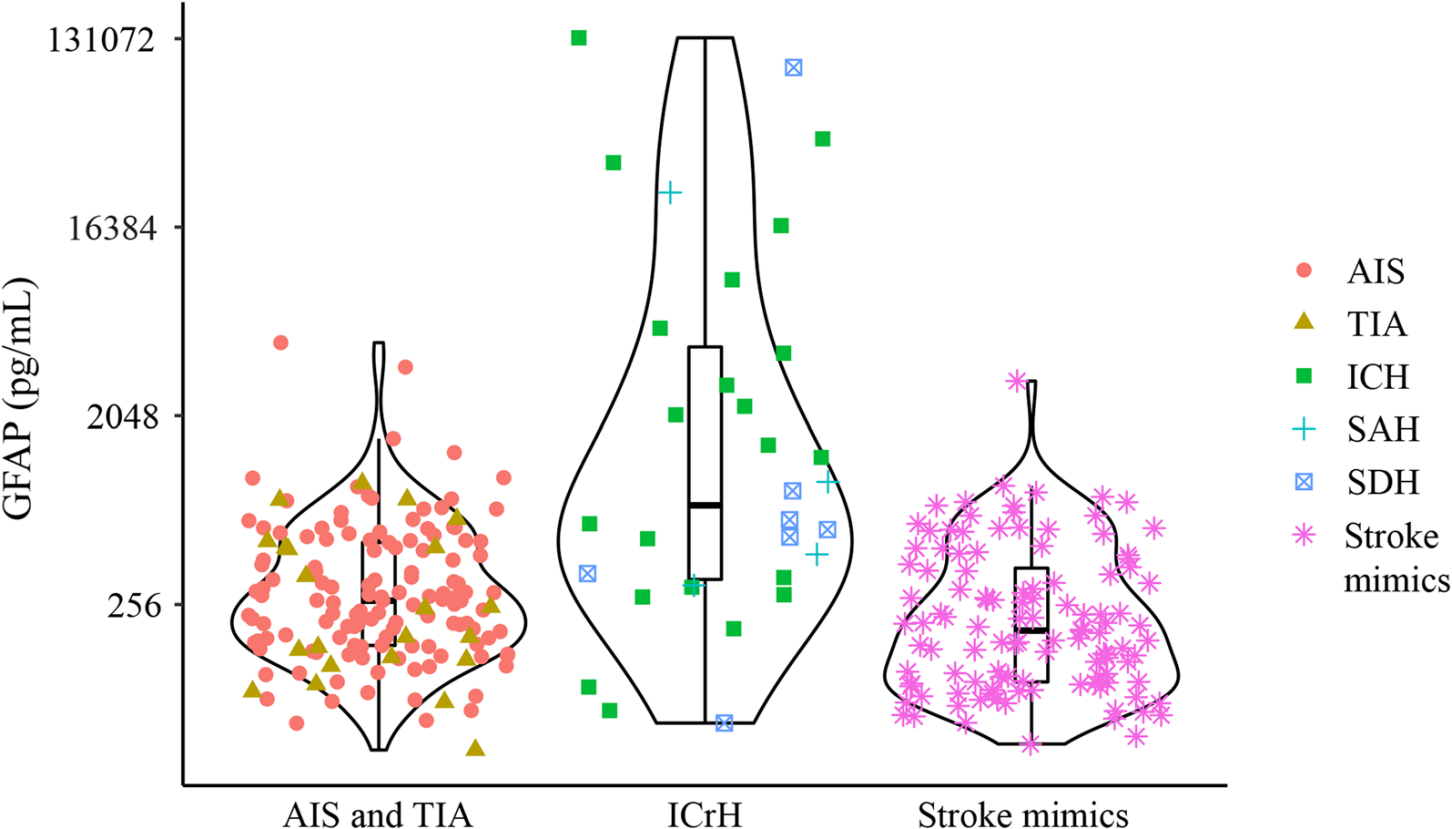
Violin plot of GFAP values by pathology. Abbreviations: *AIS* acute ischemic stroke, *GFAP* glial fibrillary acidic protein, *ICH* intracerebral hemorrhage, *ICrH* intracranial hemorrhage, *SAH* subarachnoid hemorrhage, *SDH* subdural hematoma, *TIA* transient ischemic attack

The median serum levels of GFAP differed between ICrH 775 pg/mL (IQR 331;4723) and others 230 pg/mL (IQR 145;465), and between ICH 1484 pg/mL (IQR 310;5370) and AIS 268 pg/mL (IQR 171;514). The median GFAP level for patients with LVO was 267 (IQR 157;646) pg/mL and for stroke mimics (including SDH) 203 (IQR 109;441) pg/mL (Table 1 and Fig. 3).

GFAP identified ICrH with an AUC of 0.73 (95% CI 0.62–0.84) (Table 2). A cut-off at 5369 pg/mL resulted in 25% sensitivity (95% CI 11.5–43.4) and 100% specificity (95% CI 98.6–100.0) (Table 3). A reduction in GFAP cut-off level to 838 pg/mL increased the sensitivity to 50.0% (95% CI 31.9–68.1) and decreased the specificity to 95.1% (95% CI 91.8–97.4). In this study, the cut-off for 100% specificity was used for further calculations as it isolated a group of patients with ICrH (because of absolute contraindication for tPA treatment). PreSS part 1 alone identified stroke (AIS, ICH, and SAH) and TIA with an AUC of 0.64 (95% CI 0.58–0.71) (Table 2). The predefined cut-off at ≥ 1 point yielded a 94.4% sensitivity (95% CI 89.9–97.3) and 17.4% specificity (95% CI 11.1–25.3) (Table 3).

**Table 2:** AUCs of GFAP and PreSS for identification of stroke, ICrH, and LVO

	GFAP		PreSS part 1		PreSS part 2		GFAP and PreSS^a^	
	Apparent AUC	Cross fold validated AUC	Apparent AUC	Cross fold validated AUC	Apparent AUC	Cross fold validated AUC	Apparent AUC	Cross fold validated AUC
Stroke and TIA	0.62 (0.56–0.69)	0.54 (0.47–0.60)	0.66 (0.60–0.72)	0.64 (0.58–0.71)	n/a	n/a	0.65 (0.58–0.71)	0.65 (0.59–0.71)
ICrH	0.79 (0.69–0.89)	0.73 (0.62–0.84)	0.71 (0.61–0.81)	0.69 (0.58–0.80)	0.71 (0.62–0.81)	0.66 (0.57–0.79)	0.80 (0.71–0.90)	0.71 (0.59–0.83)
LVO	0.44 (0.32–0.55)	0.35 (0.23–0.46)	0.76 (0.66–0.85)	0.72 (0.62–0.82)	0.69 (0.58–0.81)	0.66 (0.54–0.78)	0.71 (0.60–0.82)	0.67 (0.55–0.79)

*AUC* area under the receiver-operating characteristics curve, *GFAP* glial fibrillary acidic protein, *ICrH* intracranial hemorrhage, *LVO* large-vessel occlusion, *PreSS* Prehospital Stroke Score, *TIA* transient ischemic attack

^a^GFAP combined with: PreSS part 1 to identify stroke and TIA; PreSS part 1 and 2 to identify ICrH; and PreSS part 2 to identify LVO

Numbers in parentheses = 95% confidence interval

**Table 3:** Performance of GFAP and PreSS for identification of stroke, ICrH, and LVO

Stroke subtype	Sensitivity% (95% CI)	Specificity% (95% CI)	PPV% (95% CI)	NPV% (95% CI)	LR+ (95% CI)	LR− (95% CI)
**GFAP (n = 299)**						
ICrH	25.0 (11.5–43.4)^a^	100.0 (98.6–100.0)^a^	100.0 (63.1–100.0)^a^	91.8 (88.0–94.6)^a^	–	0.75 (0.61–0.92)^a^
**PreSS part 1 (n = 299) Stroke/TIA suspected if PreSS part 1 ≥ 1**						
Stroke/TIA	94.4 (89.9–97.3)	17.4 (11.1–25.3)	62.7 (56.6–68.5)	67.7 (48.6–83.3)	1.14 (1.04–1.25)	0.32 (0.16–0.66)
**PreSS part 2 (n = 268) LVO suspected if PreSS part 1 ≥ 1 and PreSS part 2 ≥ 2**						
LVO	55.6 (35.3–74.5)	77.2 (71.4–82.3)	21.4 (12.5–32.9)	93.9 (89.7–96.8)	2.43 (1.62–3.67)	0.58 (0.38–0.88)
**PreSS and GFAP combined (as used in the decision tree)**						
Step 1 Stroke and TIA (Group 4)	94.4 (89.9–97.3)	17.4 (11.1–25.3)	62.7 (56.6–68.5)	67.7 (48.6–83.3)	1.14 (1.04–1.25)	0.32 (0.16–0.66)
Step 2 ICrH (Group 1)	25.0 (11.5–43.4)^a^	100.0 (98.6–100.0)^a^	100.0 (63.1–100.0)^a^	91.8 (88.0–94.6)^a^	–	0.75 (0.61–0.92)^a^
Step 3 LVO (Group 2)	55.6 (35.3–74.5)^a^	82.4 (77.3–86.7)^a^	23.8 (14.0–36.2)^a^	94.9 (91.3–97.3)^a^	3.15 (2.06–4.81)^a^	0.54 (0.35–0.83)^a^

*GFAP* glial fibrillary acidic protein, *ICrH* intracranial hemorrhage, *LR+* positive likelihood ratio, *LR− * negative likelihood ratio, *LVO* large-vessel occlusion, *NIHSS* National Institutes of Health Stroke Scale, *NPV* negative predictive value, *PPV* positive predictive value, *PreSS* Prehospital Stroke Score, *TIA* transient ischemic attack

^a^GFAP cutoff set at 5369 pg/mL

PreSS part 2 alone detected LVO with an AUC of 0.66 (95% CI 0.54–0.78) (Table 2). The predefined cut-off at ≥ 2 points and a positive PreSS part 1 produced a sensitivity of 55.6% (95% CI 35.3–74.5) and a specificity of 77.2% (95% CI 71.4–82.3) (Table 3).

**Fig. 4:**
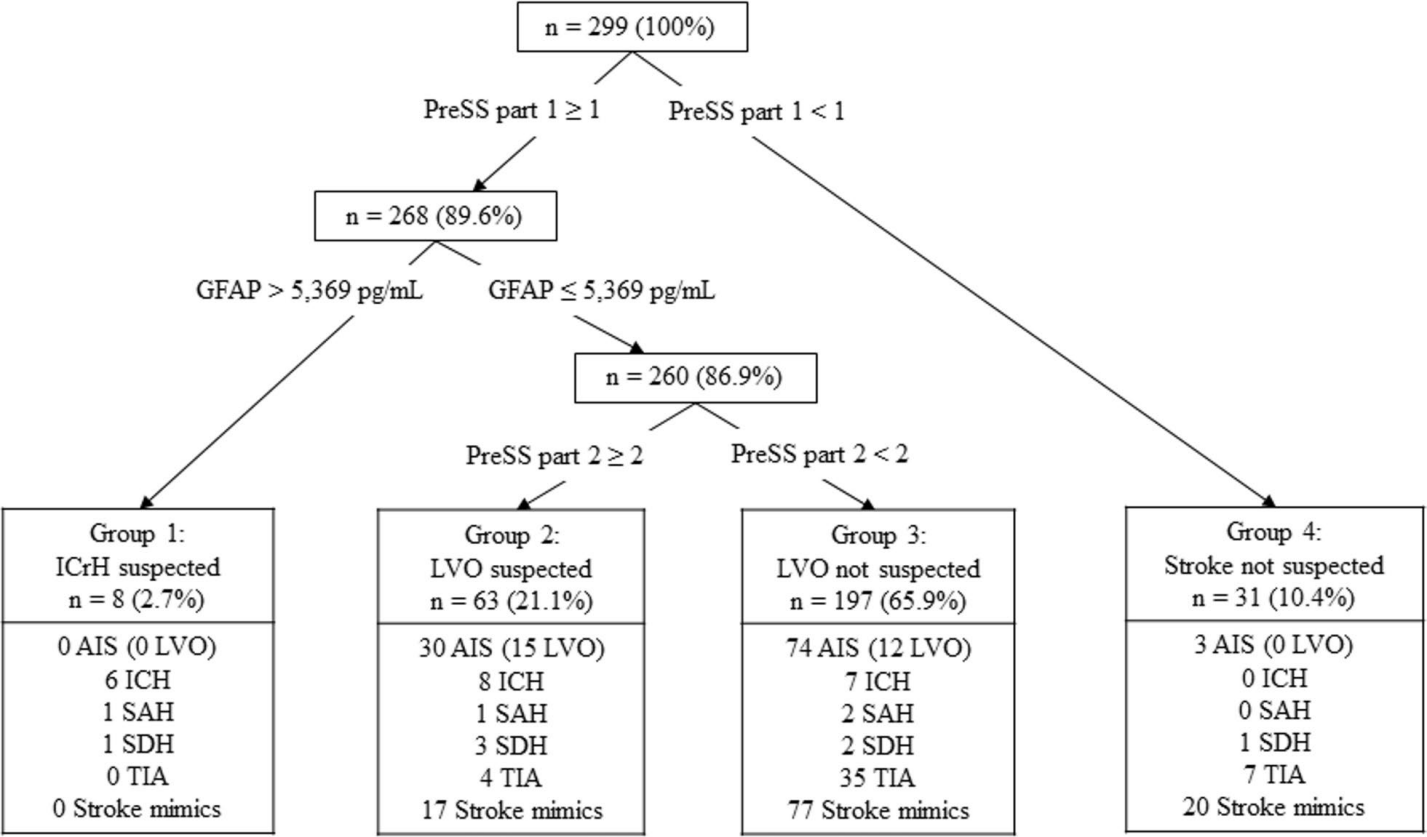
Decision tree for stroke subtype identification using GFAP cut-off 5369 pg/mL and PreSS. Abbreviations: *AIS* acute ischemic stroke, *GFAP* glial fibrillary acidic protein, *ICH* intracerebral hemorrhage, *LVO* large-vessel occlusion, *PreSS* Prehospital Stroke Score, *SAH* subarachnoid hemorrhage, *SDH* subdural hematoma, *TIA* transient ischemic attack

PreSS and GFAP values combined did not increase the performance for stroke subtype differentiation markedly over either GFAP or PreSS separately (Table 2). Combining PreSS and GFAP in a decision tree divided the population in a stepwise manner into four groups (Fig. 4). Step 1: PreSS part 1 excluded 31 patients (AIS n = 4, SDH n = 1, TIA n = 6 stroke mimics n = 20) by a result of 0 points (Group 4, in Fig. 4). The four patients with AIS had low NIHSS scores of 0, 0, 1, and 2 points. Step 2 using a GFAP cut-off of 5369 pg/mL identified ICrH with a sensitivity of 25.0% (95% CI 11.5–43.4) and a specificity of 100.0% (95% CI 98.6–100.0) (Table 3) and isolated a group of patients with ICrH consisting of ICH n = 6, SAH n = 1, and SDH n = 1 (Group 1 in Fig. 4). In Step 3 (the final step), PreSS part 2 identified two groups of patients: a group with positive PreSS part 2 (group 2) and a group with negative PreSS part 2 (Group 3 in Fig. 4). The stepwise division and the predefined cut-off of PreSS part 2 yielded a sensitivity of 55.6% (95% CI 35.3–74.5) and a specificity of 82.4% (95% CI 77.3–86.7) for LVO detection. The combination of GFAP and PreSS therefore resulted in an increased specificity (from 77.2 (95% CI 71.4–82.3)) but only a very modest increase in the cross-validated AUC from 0.66 (95% CI 0.54–0.78) to 0.67 (95% CI 0.55–0.79) for LVO detection. Patients with LVO in Group 2 (suspected LVO) had a median NIHSS of 14 (IQR 12;20), whereas patients with LVO in Group 3 (without suspicion of LVO) had a median NIHSS of 5 (IQR 2;8). The proportion of AIS caused by LVO in Group 3 was 12.6% (95% CI 6.7–21.0); in Group 2, 46.9% (95% CI 29.1–65.3). The pretest probability for LVO among AIS in the entire cohort was 20.6% (95% CI 14.0–28.6).

As sensitivity analyses, we calculated the performance of the classical CPSS (including the items face, arm and speech, equivalent to the face-arm-speech test (FAST)) instead of PreSS part 1 and the full NIHSS dichotomized (0–5 and ≥ 6 points) instead of PreSS part 2 with NIHSS ≥ 6 as an indicator for LVO. Integrated in the decision tree, the classical CPSS used in step 1 to identify stroke or TIA had an AUC of 0.61 (95% CI 0.56–0.67) with a sensitivity of 83.2% (95% CI 77.1–88.3) and a specificity of 38.6% (95% CI 29.6–48.2). The performance of GFAP in step 2 to identify ICrH was unchanged. To identify LVO in the final step 3, NIHSS had an AUC of 0.67 (95% CI 0.58–0.77) and the chosen cut-off (NIHSS ≥ 6) resulted in a sensitivity of 73.1% (95% CI 52.2–88.4) and a specificity of 61.6%. PreSS part 1 was slightly superior to the classical CPSS measured by AUC (0.64 versus 0.61). PreSS part 2 had a similar performance as the full NIHSS (AUC of 0.67 for both scales).

GFAP could not differentiate stroke mimics (including SDH) from AIS shown by an AUC of 0.47 (95% CI 0.40–0.55) or stroke mimics (including SDH) from stroke and TIA with an AUC of 0.54 (95% CI 0.47–0.60).

## Discussion

In patients with symptoms lasting up to 4.5 h, we used GFAP to identify ICrH and PreSS to identify stroke and LVO. Using GFAP and PreSS together in a decision tree may improve prehospital differentiation of stroke, and thereby improved transport—and treatment strategies.

First, PreSS part 1 (0 points) excluded a group of patients with a low probability of stroke with only few “false negative” AIS cases, all of whom had a very low NIHSS. This group could be admitted to the nearest emergency department. Second, using GFAP we were able to isolate a group of patients with ICrH. This may be used to introduce very early treatment in the prehospital setting such as hemostatic therapy or lowering of high blood pressure due to the 100% specificity of GFAP to exclude other diagnoses (e.g., AIS). Optimal treatment has yet to be fully clarified for patients with ICrH, but acute antihypertensive treatment within 2 h are currently recommended [3]. To enable such prompt treatment, prehospital identification is pivotal in most cases. Excluding a group of ICrH also increased the specificity of PreSS part 2 for subsequent LVO identification.

In the final step of the decision tree, two groups of patients were identified by PreSS part 2; one group (PreSS part 2-negative) in which the probability of LVO was nearly halved, and one group (PreSS part 2-positive) in which the probability of LVO was more than twice as high as the pretest probability. Furthermore, the median NIHSS was higher in the test-positive group than in the test-negative group (14 versus 5). PreSS part 2 identified not only a group of patients with a high probability of LVO but also patients with LVO with the highest level of neurological impairment. This group of patients may benefit from direct triage to a CSC for more rapid EVT evaluation.

Based on the sensitivity analysis, the performance was slightly lower when using the classical CPSS compared with the PreSS part 1 for detecting stroke and TIA, and the performance was equal when using the full NIHSS compared with PreSS part 2 for detecting LVO. The performance of PreSS part 1 (the classical CPSS without additional items) showed an increased sensitivity at the expense of a lower specificity. Highest possible sensitivity is desired in our stroke management because all test-positive patients are evaluated for direct stroke center admission by a telephone conference call to an on-duty stroke neurologist. This conference call has previously been shown to increase the specificity markedly with only a small reduction in sensitivity [14]. With an equal performance of PreSS part 2 and NIHSS, the simple dichotomized PreSS part 2 could be preferred because of its high feasibility [13].

Other studies examining biomarkers and stroke scales in combination are few. GFAP and D-dimer measured with ELISA have been combined with symptom-based stroke scales for LVO identification [26]. Adding biomarkers (GFAP and D-dimer) to a prehospital stroke scale was associated with a high AUC of 0.95 (95% CI 0.91–1.00). However, the performance of the best stroke scale in the study (The Field Assessment Stroke Triage for Emergency Destination) already identified LVO with an AUC of 0.91 (95% CI 0.86–0.95), which is higher than in comparable studies and may be attributed to a degree of selection bias (LVO accounted for 35% of all AIS) [26].

In our study, the increase in AUC achieved by combining GFAP and PreSS part 2 was more limited (Table 2). This may be due to the use of a single biomarker, an unselected patient cohort, and five-fold cross validation. The patient cohort in this study consisted of 38% stroke mimics and GFAP could not differentiate these patients from stroke and TIA patients.

PreSS part 1 excluded 20 patients from further stroke suspicion. The low number is caused by the low specificity of PreSS part 1. We did not combine this initial step with a telephone conference. The relatively high proportion of stroke mimics, seen in this study, has also been reported in other studies from Denmark and Norway with similarly organized stroke care [14, 35]. The high proportion of stroke mimics emphasizes that the included patients represent a realistic unselected prehospital population of suspected stroke patients.

The ability of GFAP to differentiate between AIS and hemorrhagic stroke (ICH and SAH) is well established in selected cohorts consisting only of the two pathologies in question with or without healthy controls [36–38]. Other studies included patients with a NIHSS ≥ 3–4 points that, apart from the inclusion of a few stroke mimics, produced cohorts also consisting of mainly AIS and ICH [23, 25, 39]. Using these selective inclusion criteria with a minimum required NIHSS of ≥ 3, Mattila et al. measured GFAP with the SIMOA technique and found that cut-off values ruled out most hemorrhagic strokes, isolating a proportion of the AIS patients [40]. Interestingly, this performance increased when adding the release rate of GFAP based on two early drawn blood samples. However, in the study by Mattila et al., two out of 59 patients with ICH remained below the cut-off and in the core GFAP range of patients with AIS at the best performance. In our study, we were unable to reproduce findings showing that GFAP was able to rule out hemorrhagic strokes in our patient cohort with all NIHSS scores included. As seen in Fig. 3, the GFAP values for patients with ICrH were evenly scattered in the low-value area—like the AIS patients. The lowest GFAP values for ICH and AIS were equally low (79.4 ng/mL and 87.8 ng/mL, respectively). This affects the prospects of prehospital treatment with tPA based on a hemorrhagic stroke rule-out by GFAP used in the studies by Mattila et al. and Bustamante et al.

The diagnostic performance of both symptom-based stroke scales and GFAP tends to increase as the inclusion of patients becomes increasingly selected. In the study by Mattila et al., the performance increased significantly when NIHSS inclusion criteria were tightened from ≥ 3 to > 8 points. Our study included patients in the prehospital phase based only on a suspicion of stroke, thus providing results for an unselected patient group relevant to prehospital stroke management [41]. Furthermore, we reported cross-fold validated AUCs providing performance estimates with a lower risk of bias than seen with other methods.

Moreover, the treatment strategy in Norway is liberal when it comes to initiation of intravenous tPA treatment where also AIS patients with low NIHSS (< 3 point) receives tPA [42]. Hence, selected cohorts based on NIHSS does not include all stroke patients of interest to our stroke care.

If point-of-care-testing for GFAP becomes available, preferable on whole blood, the approach applied in the present study using a decision tree may help divide patients into groups for whom specific prehospital management may be applied including very early treatment of patients with ICrH. Also, the addition of other biomarkers to the decision tree developed in this study might improve stroke identification and stroke subtype differentiation in the prehospital setting. Our study and the study by Mattila et al. provide evidence that the highly sensitive SIMOA technique may be used to measure GFAP in patients with shorter median times from symptom onset to blood collection, than the optimal window of 2–6 h reported in previous studies [16].

In a previous study, patients suffering from SAH had GFAP levels below the cut-off value. [19]. In our study, patients suffering from all diagnoses within the ICrH category (ICH, SAH and SDH) had GFAP values in both the upper and lower end (Fig. 3).

A limitation to this study was that the PreSS assessment was not performed by the prehospital personnel. PreSS was constructed retrospectively from the full NIHSS performed by anesthesiologists and paramedics in the MSU/ambulance or by on-call stroke physicians at hospital admission. Even so, prospective assessment with PreSS would likely be comparable to the retrospectively calculated PreSS as the full NIHSS was performed by prehospital personnel for the majority of the included patients.

## Conclusion

The combination of a symptom-based stroke scale for stroke and LVO identification and GFAP for ICrH detection in a decision tree might enable prehospital differentiation of ICrH, AIS, LVO and stroke mimics. The differentiation can facilitate triage to the correct levels of care and reduce time from symptom onset to initiation of diagnose-specific treatments.

## Data Availability

The data are available from the corresponding author upon reasonable request.

## Abbreviations

AIS: Acute ischemic stroke
AUC: Area under the receiver operating characteristic curve
BA: Basilar artery
CI: Confidence interval
CPSS: Cincinnati Prehospital Stroke Scale
CSC: Comprehensive stroke center
CT: Computed tomography
ELISA: Enzyme-linked immunosorbent assay
EVT: Endovascular treatment
FAST: Face-arm-speech test
GFAP: Glial fibrillary acidic protein
ICA: Internal carotid artery
ICH: Intracerebral hemorrhage
ICD-10: International Classification of Diseases, 10th Version
ICrH: Intracranial hemorrhage (ICH, SAH and SDH)
IQR: Interquartile range
LR−: Negative likelihood ratio
LR+: Positive likelihood ratio
LVO: Large-vessel occlusion
MRI: Magnetic resonance imaging
MSU: Mobile stroke unit
NIHSS: The National Institutes of Health Stroke Scale
NPV: Negative predictive value
PPV: Positive predictive value
PreSS: Prehospital Stroke Score
PSC: Primary stroke center
SAH: Subarachnoid hemorrhage
SD: Standard deviation
SDH: Subdural hematoma
SIMOA: Single-molecule array
TIA: Transient ischemic attack
tPA: Tissue plasminogen activator
Treat-NASPP: Treat Norwegian Acute Stroke Prehospital Project
VA: Vertebral artery
